# Oncology Drug Shortages in America

**DOI:** 10.6004/jadpro.2012.3.2.4

**Published:** 2012-03-01

**Authors:** Wendy H. Vogel, Robert S. Ervin

**Affiliations:** From Kingsport Hematology Oncology Associates, Kingsport, Tennessee

The issue of drug shortages is only one of the many crucial concerns facing oncology advanced practitioners (APs) in the United States today. However, it may be the most critical issue affecting short- and long-term patient outcomes. Short-term outcomes may be affected by treatment delays, treatment with less effective alternatives, and increased toxicity. Long-term outcomes may be negatively impacted by the delay in or suspension of important clinical trials. The challenge to APs during times of drug shortages is to ensure that patient treatment is efficacious and safely continued. Both deliberate preparation and excellent communication are required for seamless care. The AP is a key member of the health-care team that must address drug shortages.

## Background

Drug shortages have increased tremendously over the past 6 to 10 years. The Drug Information Service of the University of Utah Hospitals and Clinics began tracking drug shortages in 2001 (Fox & Tyler, 2003). This group noted more than a threefold increase in the number of drug shortages from 2005 to 2010. These shortages are not limited to oncology drugs, although the oncology community has been one of the hardest hit. There are shortages of sterile injectables such as antibiotics, electrolytes, nutritional agents, supportive care medications, and surgical pharmaceuticals (US Food and Drug Administration [FDA], 2011a). The largest number of drug shortages was reported in 2010, but the figures for 2011 may well have superseded this number (FDA, 2011b). Most of the shortages are for generic drugs. Oncology (including pediatric oncology) and anesthesiology are two areas that have been severely affected. A recent survey by the Association of Community Cancer Centers (Eastman, 2011) found that 94.4% of survey participants had experienced an oncology drug shortage.

Table 1 lists some oncology drugs that have been in short supply during the past year. These 22 drugs are on the FDA shortage list and are commonly used in oncology practices (FDA, 2011a). Even though these agents are older, primarily generic agents, they are critical in many standard regimens (in often curable disease states) and may not have an acceptable alternative agent. Shortages have affected many different types of cancer treatment, including, but not limited to, breast, colorectal, lymphoma, sarcomas, testicular, and several childhood cancers. These shortages affect clinical trials as well, causing protocol deviations, enrollment delays, and compromised data.

**Table 1:**
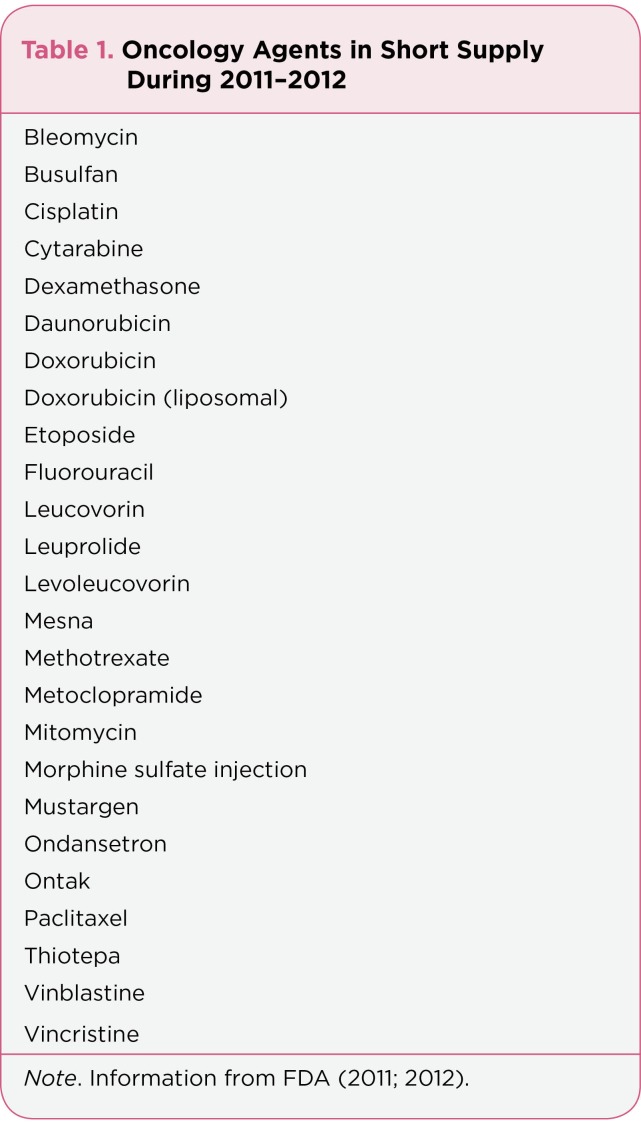
Table 1. Oncology Agents in Short Supply During 2011–2012

## Causes and Other Compounding Factors

The causes of drug shortages in the United States are varied. It is at times difficult to ascertain the cause of some shortages, but most can be attributed to one of the factors listed in Table 2. One of the most complex causes is the fact that the majority of the raw materials used in US pharmaceuticals come from other countries (Fox et al., 2009). Problems in these countries, such as wars, natural disasters, or adverse climate conditions, may affect production in the United States. Manufacturer delays or cessation of production of a particular agent may cause a drug shortage, especially when a limited number of companies produce that agent. Because manufacturers are not bound by any rule or regulation to report the actual cause of a shortage, the health-care industry is left merely guessing while the stock prices of the manufacturers dip only slightly in uncertainty. Rumors abound in this environment, and this adds to the pitfalls of allocation and stockpiling.

**Table 2:**
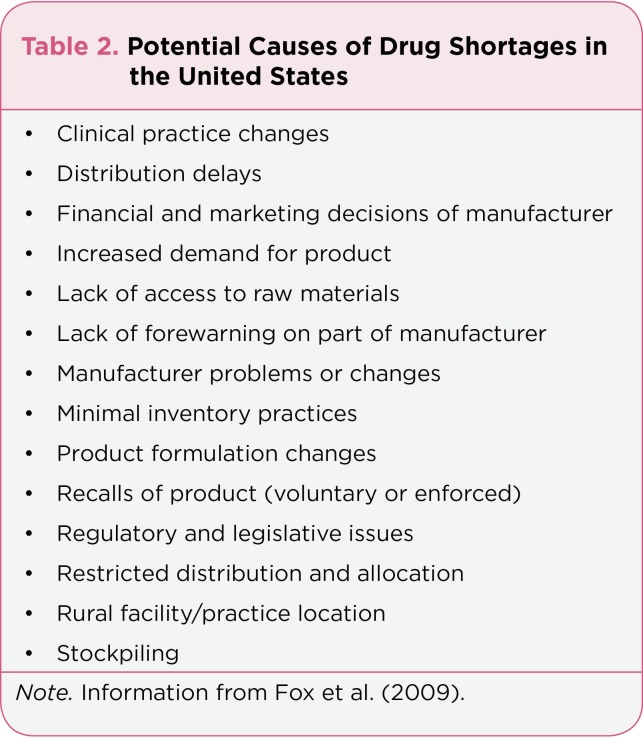
Table 2. Potential Causes of Drug Shortages in the United States

The US Food and Drug Administration (FDA) may force the shutdown of manufacturing a product due to noncompliance with current good manufacturing practices (Fox et al., 2009). As it may not be financially feasible for some manufacturers to bring production into compliance, production may be permanently halted. The FDA does not have the authority to force a manufacturer to continue production, even if the product is deemed medically necessary and there are no other manufacturers. Bringing production into compliance may be time-consuming for both the manufacturer and the FDA, further delaying production.

The Medicare Modernization Act is often cited as a cause of the drug shortage crisis (Gatesman & Smith, 2011). This act set reimbursement at the average sales price plus a 6% markup. This significantly decreased profits made by oncology practices and health-care facilities from the sale of oncology drugs. This may have steered many practices toward more costly nongeneric equivalents in order to maintain profitability.

Some drugs have restricted distribution or allocation processes; manufacturers may limit the availability of specific drug products to certain end users/suppliers (Fox et al., 2009). The inventory practice of keeping a minimal supply of drugs in stock only after need is identified may contribute to drug shortages, especially when the shortage is unexpected. Stockpiling (hoarding) of drugs when a shortage is expected can exacerbate this scarcity. Delivery delays and difficulty "borrowing" from neighboring facilities due to a remote location of a facility or practice may cause drug shortages.

A growing problem in the United States is the so-called "gray market" (also called the open market or alternative distribution). Gray-market distributors obtain drugs that are in short supply for the purpose of selling them to end users for a profit, often at exorbitant prices. Gray markets may also cause additional concerns such as refusal of returns or refunds for expired or unused product. It may also be impossible to determine the drug’s source (or pedigree), as these suppliers may not be able to provide documentation of authenticity (Institute for Safe Medication Practices [ISMP], 2011). Not only do these dubious distributors profit from such shortages, but reputable companies enjoy explosive business in both volume and pricing when a branded alternative is available for a decades-old generic product. Reports of up to 60-fold price increases for these already more expensive products essential to continued therapy have been reported (Goldberg, 2009).

## Far-Reaching Negative Effects

In addition to the threat of compromised outcomes, patients may suffer from other potential negative effects of drug shortages (Table 3). One growing concern is the increased potential for a medication error. Over one-third of respondents to a 2010 ISMP survey of more than 1,800 health-care practitioners reported that their facility had experienced a "near miss" of an error directly related to a drug shortage that could have harmed a patient (ISMP, 2010a). Some errors reported included dosing errors when alternative strengths or drugs were utilized and incorrect reconstitution occurred.

**Table 3:**
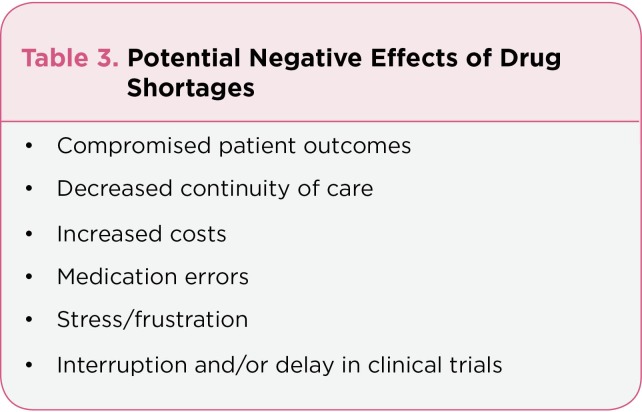
Table 3. Potential Negative Effects of Drug Shortages

There are also negative financial effects due to drug shortages. A significant amount of time may be spent in the procurement of drugs and the search for an acceptable alternative. These activities are most often performed by highly compensated employees such as registered nurses, APs, pharmacists, physicians, and practice managers (Kaakeh et al., 2011). The cost of this time is not recoverable. Often the cost of the procured or alternative drug is much higher, increasing the cost of care.

Many APs, pharmacists, and physicians express frustration and increased stress from the management of drug shortages (Nelson, 2011). At times, practitioners are placed in a position of rationing drugs—making difficult decisions about who to treat with the little drug that is available to the practice. Knowing that there is a potentially curative therapy available, but being unable to treat a patient in a timely manner, is a disturbing prospect. Stockpiling or "hoarding," as mentioned previously, is a practice that may deny a patient an essential treatment in another practice, but it is a tempting prospect to any practitioner who has had to deny a patient treatment due to unavailability or is aware of possible patients in the near future. These shortages are bringing a whole new ethics challenge to health care, an industry that has always held itself to the highest of standards.

## Steps to Prepare for Challenges Ahead

Advanced practitioners in oncology must be aware of impending drug shortages and prepared to address this issue with patients and staff. Efficient preparation for and effective management of drug shortages will decrease the risk of potential problems that may compromise patient outcomes. Table 4 lists some "Do’s" and Don’ts" in the management of drug shortages. Ideally, one (or two) team members should be determined to be the "point person(s)" (Fox et al., 2009). This person should regularly assess for signs of a potential shortage such as partially filled orders, a particular drug strength becoming difficult to obtain, or limited manufacturer stock (Fox et al., 2009; ISMP, 2010b). This person should regularly search resource websites for information. The point person should also facilitate team meetings and strategy planning.

**Table 4:**
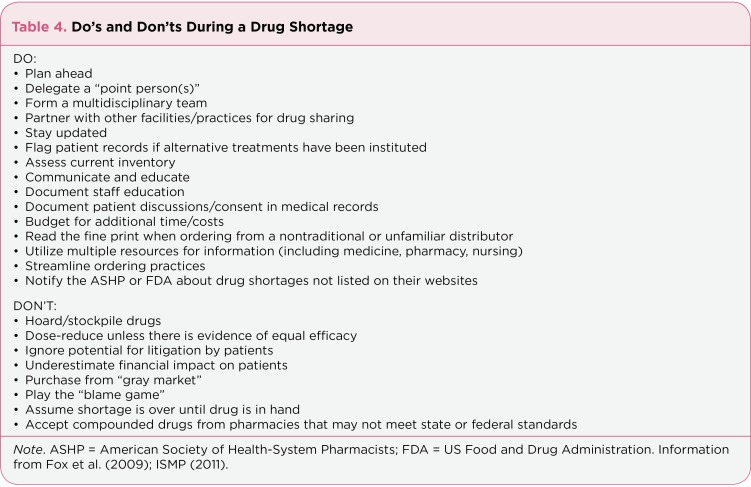
Table 4. Do’s and Don’ts During a Drug Shortage

As noted in Table 5, preparation for a drug shortage includes several steps. Because each facility’s method of handling a drug shortage will differ according to the type of practice setting and the practice’s preference, these steps are given as a suggested structure to be applied as needed to each setting. Currently, there are no evidence-based guidelines for handling drug shortages (Tobin, 2011). Gathering a multidisciplinary team will ensure that all potential issues related to the drug shortage are addressed. This team could include (but is not limited to) medicine, nursing, pharmacy, financial aid, billing/reimbursement, purchasing, patient education, patient financial resources, and scheduling staff.

**Table 5:**
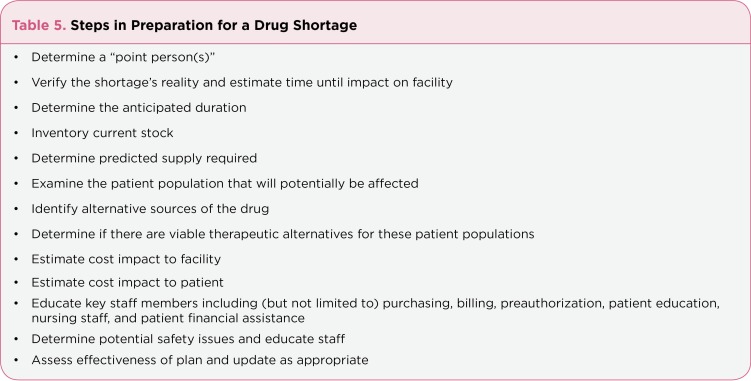
Table 5. Steps in Preparation for a Drug Shortage

## Gather Available Information About the Shortage

One of the earliest steps in preparation for a drug shortage is to verify the shortage’s reality and anticipate the time until the shortage could impact the facility. Two excellent resources to assess the validity of a reported shortage are noted in Table 6 along with other resources. The FDA and the American Society of Health-System Pharmacists (ASHP) both have regularly updated listings of reported drug shortages. The FDA site gives an explanation of the shortage cause if known, as well as the anticipated release of the drug. The ASHP site provides similar information in addition to links to related shortages of similar medication classes. If there is a shortage and it is not listed on one of these sites, instructions are available on these sites to report this shortage. Other resources for drug shortage information include professional electronic mailing lists, drug wholesalers, pharmaceutical representatives, and the manufacturers themselves.

## Examine Resources and Needs

Information gathered may assist in predicting the duration of the drug shortage after examining the current inventory in stock. It is important to estimate what a practice’s required supply will be. Examining previous months’ usage of the drug will assist in this estimation. Patient populations that will utilize the drug should be determined. Following this self-examination, alternative sources of the drug should be identified. Regional oncology practices may work together in sharing limited supplies. It is unethical for a drug to go out of date on one practice’s shelf when another nearby practice is in need. Facilities may need to determine appropriate alternative suppliers, including in-house compounding ability (ISMP, 2010b). Clinically appropriate uses of the drug in shortage should be determined. Assess how this drug is being used in the facility. Research the most effective dose regimens that will treat patients appropriately, but limit waste to a minimum.

**Table 6:**
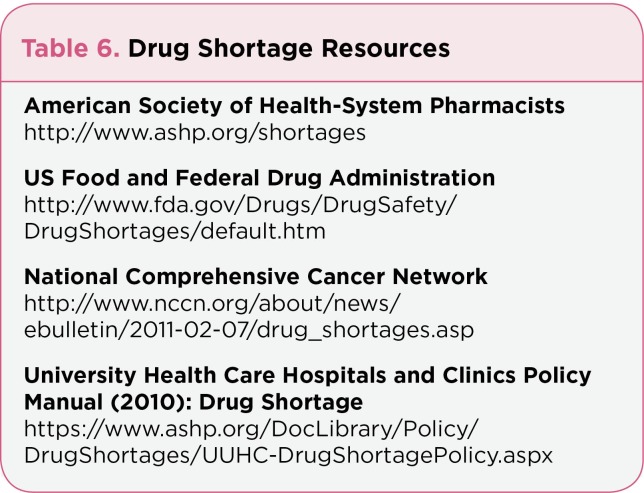
Table 6. Drug Shortage Resources

## Determine Viable Alternatives

Viable therapeutic alternatives for patient populations utilizing the drug should be determined. Ideally, a multidisciplinary team of oncologist/hematologists, advanced practitioners, nurses, and pharmacy representatives should be involved in examination of the literature and accepted treatment guidelines. Professional societies such as the American Society of Clinical Oncology, the Oncology Nursing Society, the American Society of Hematology, and the Hematology/Oncology Pharmacy Association, and the ASHP may provide useful information. A difficult dilemma occurs if there is no viable alternative regimen. Then the challenging task of rationing available medication ensues. Prescribing clinicians may choose to preferentially treat those patients that are being treated adjuvantly (anticipating a cure) vs. those who are being treated in a metastatic setting. Another dilemma occurs when an alternative regimen may be equal in efficacy but the alternative regimen has more undesirable side effects. When choosing an alternative regimen, the "point person" should research the availability of the alternative drug and anticipate any potential shortages based on increased demand for this option.

The research team may also be a key part of the multidisciplinary team. There are unique issues related to drug shortages in clinical trials. According to the Coalition of Cancer Cooperative Groups (2011), about one-half of all active cooperative group cancer clinical trials have at least one drug on the shortages list. When a drug utilized in a clinical trial is in short supply, enrollment in the trial may be compromised, thus increasing the amount of time required to accrue enough patients to be statistically significant. Trial start dates may be delayed due to uncertainties over availability of study drugs (National Coalition for Cancer Research, 2011). Some patients may have to be removed from a study because of a drug shortage. These problems will ultimately slow the progress of scientific research (National Coalition for Cancer Research, 2011). Research sites must ensure a drug’s availability prior to enrollment. The sponsor of the trial and the principal investigator will be queried regarding decisions about protocol deviations (such as drug substitution) or alternative procedures. For many trials, substitutions will not be permitted. In addition, if protocol deviations are permitted or changes/substitutions made, then this must be reported to the Internal Review Board, who may need to review protocol changes and/or issues related to consent or patient safety.

## Educate All Staff on Required Changes

Alternative therapies should be selected early so that all staff may be adequately educated on required changes. Collaboration with purchasing and billing staff will be necessary to assess the financial impact to the facility. Cost to the facility is not only the cost of the drug in shortage or the selected alternative treatment, but also the cost of staff time in terms of alternative treatment research, drug procurement, shortage team meetings, education, and additional patient face-to-face interaction. Ultimately, the burden related to drug shortages is borne by patients, not just in terms of outcomes but also in monetary costs (Melton, 2011). The financial impact to the patient must always be considered. Personnel who preauthorize treatments or assist patients in obtaining financial assistance for medications will also need to be informed about any impending changes. This is particularly important in an era of dwindling financial resources, increasing insurance restraints, and a large number of uninsured or underinsured patients.

## Examine Safety Concerns

The risk of any potential safety concerns should be examined. The ISMP survey on drug shortages reported that about 20% of practitioners surveyed had "near-misses, errors, and adverse outcomes" related to drug shortages (ISMP, 2010a). Drug dosages, concentrations, infusion rates, bioavailability, activity, preparation methods, and routes of alternative or substituted medications should be clarified (Melton, 2011). Potential side effects and toxicities of alternative treatments should be reviewed. Any sound- or look-alike issues with the alternative medication should be noted (ISMP, 2010b). Research into the literature should be done to see if any specific errors have been associated with the alternative treatment. Any approved Risk Evaluation and Mitigation Strategies (REMS) programs for alternative medications should be clarified and instituted as appropriate.

## Maintain Communication Among Staff and Patients

A critical step in effectively managing a drug shortage is adequate communication between all team members involved, including the patient. Use of all effective means of communication (verbal announcements, e-mail, signage, electronic medical record alerts, etc.) will ensure that everyone is aware of any changes (Fox et al., 2009). Certain team members, such as ordering clinicians, pharmacists, and nursing staff, will need daily updates during a critical drug shortage.

Patients may experience some psychological issues secondary to drug shortages; these may include fear, anxiety, and distrust of the medical system (Melton, 2011). Patients must receive a consistent message about drug shortages and understand how their health-care team is proactively addressing the challenge. Effective communication with patients about proactive measures to manage the drug shortage fosters greater trust and respect instead of giving the "blame game" explanation (blaming the system, the pharmaceutical company, or the government). Knowing that there is a "plan B" in case of a shortage will often relieve patient fears. Reassurance should be reasonably offered. There is the potential for litigation by patients who suffer adverse events from alternative treatments who believe a poor outcome was due to a treatment change or delay (Fox et al., 2009).

## Assess Effectiveness

Ongoing assessment of any alternative treatment measures by all team members is necessary. Patient tolerance, adverse events, efficacy, and financial impact are all elements to consider. Any errors related to alternative treatments secondary to the drug shortage should be scrutinized. Periodic discussion of the effectiveness of the drug shortage response plan should ensue, and any adjustments to the process should be instituted as deemed necessary.

## The Role of the Advanced Practitioner

The AP in oncology is continually challenged to provide uninterrupted, safe, and efficacious care. Successful management of drug shortages is complex and best addressed by a multidisciplinary team. The AP can play a key leadership role on this team and in the development of the processes by which his or her facility handles drug shortages. The AP may serve as a liaison between the multidisciplinary team and staff and between the team and patients. The role of the AP must also include involvement in any legislative processes affecting drug shortages. In a crisis such as the shortage of oncology drugs, the AP perspective is invaluable.
